# Fingerprinting Metabolic Activity and Tissue Integrity of 3D Lung Cancer Spheroids under Gold Nanowire Treatment

**DOI:** 10.3390/cells11030478

**Published:** 2022-01-29

**Authors:** Hadi Hashemzadeh, Ali Hamad Abd Kelkawi, Abdollah Allahverdi, Mario Rothbauer, Peter Ertl, Hossein Naderi-Manesh

**Affiliations:** 1 Nanobiotechnology Department, Faculty of Biosciences, Tarbiat Modares University, Tehran 14115-111, Iran; hadi.hashemzadeh@modares.ac.ir (H.H.); ali.hd1987@gmail.com (A.H.A.K.); 2 Biophysics Department, Faculty of Biosciences, Tarbiat Modares University, Tehran 14115-111, Iran; a-allahverdi@modares.ac.ir; 3 Faculty of Technical Chemistry, Vienna University of Technology (TUW), Getreidemarkt 9/163-164, 1060 Vienna, Austria; mario.rothbauer@tuwien.ac.at; 4 Orthopedic Microsystems Group, Karl Chiari Lab for Orthopedic Biology, Department of Orthopedics and Trauma Surgery, Medical University of Vienna, 1090 Vienna, Austria

**Keywords:** spheroid, metal nanoparticles, toxicity test, cancer, cell culture techniques

## Abstract

Inadequacy of most animal models for drug efficacy assessments has led to the development of improved in vitro models capable of mimicking in vivo exposure scenarios. Among others, 3D multicellular spheroid technology is considered to be one of the promising alternatives in the pharmaceutical drug discovery process. In addition to its physiological relevance, this method fulfills high-throughput and low-cost requirements for preclinical cell-based assays. Despite the increasing applications of spheroid technology in pharmaceutical screening, its application, in nanotoxicity testing is still in its infancy due to the limited penetration and uptake rates into 3D-cell assemblies. To gain a better understanding of gold nanowires (AuNWs) interactions with 3D spheroids, a comparative study of 2D monolayer cultures and 3D multicellular spheroids was conducted using two lung cancer cell lines (A549 and PC9). Cell apoptosis (live/dead assay), metabolic activity, and spheroid integrity were evaluated following exposure to AuNWs at different dose-time manners. Results revealed a distinct different cellular response between 2D and 3D cell cultures during AuNWs treatment including metabolic rates, cell viability, dose–response curves and, uptake rates. Our data also highlighted further need for more physiologically relevant tissue models to investigate in depth nanomaterial–biology interactions. It is important to note that higher concentrations of AuNWs with lower exposure times and lower concentrations of AuNWs with higher exposure times of 3 days resulted in the loss of spheroid integrity by disrupting cell–cell contacts. These findings could help to increase the understanding of AuNWs-induced toxicity on tissue levels and also contribute to the establishment of new analytical approaches for toxicological and drug screening studies.

## 1. Introduction

The global trend towards three-dimensional (3D) in vitro cell cultures systems is partially driven by the fundamental biological changes, such as gene/protein profiles and cell signaling pathways, that take place when moving from classical 2D cultures to 3D cell assemblies [1,2,3]. Various 3D cell culture models exist and are classified as scaffold-free and with scaffold cell culture methods. Among the scaffold-free cell assemblies, 3D multicellular spheroid technology is predominantly used in academia and industry [3,4,5,6]. Here the inherent ability of cells to aggregate with neighbor cells and produce ECM that further promotes adhesion is exploited to generate spherical spheroids in low-attachment surfaces [7]. As a result, spheroid technologies, which are known to better reflect the tissue-specific microenvironment, are mainly used in the pharmaceutical screening applications [3,8]. The multicellular tumor spheroid (MCTS) model is able to mimic avascular tumors, in which proliferative cells are almost located at the surrounding zone of the spheroid and necrotic cells are located at the core zone. In addition, oxygen (O_2_), pH and nutrient gradients inside of a MCTS are similar to the in vivo solid tumors [9]. These 3D cell culture models are introduced into industrial processes as powerful systems with massive translational potential including stem cell research, cancer research, drug discovery, cell-based biosensors, and toxicological effects studies [10,11,12,13,14,15]. Recapitulation of in vivo morphology, mimicry of tissue architecture, good cell connectivity such as cell–cell and cell–matrix interactions and gene and protein expression profile similarity with physiological microenvironment, provide 3D cell culture models with a predictive perspective on physiological tissue behavior under drug treatment. Nevertheless, traditional two-dimensional cell culture approaches are still being used as a route for the risk assessment of nanomaterials and chemical agents [16,17,18,19]. While 2D cell culture platforms will remain an attractive research strategy to investigate basic cellular behaviors [20,21], these simple two dimensional in vitro cell-based assays are not suited to address more complex biological questions where cellular responses, cell-to-cell, and cell-to-matrix interactions are governed by the tissue-specific architecture [17,22,23,24].

This aspect is particularly important by nanomaterial–biology interactions where the uptake, transport, and distribution can be directly linked to the shape, composition, and size of the tissue structure [25]. Hence, the requirement for high-throughput and cost-effective preclinical cell-based assays for assessing the risk of nanoparticles and nano-drug screening studies needs to also involve functional 3D-tissue structures [1,2,21] that can bridge the gap between in vivo and in vitro models [6,26]. Here, three-dimensional spheroid models that are generated either by using commercial microplates, microfluidics systems [4] are ideally suited to address nanomaterial–biology interactions that require adequate cell-to-cell interactions and appropriate cell signaling events [3,27,28].

Since nanotechnology-based products and their applications in various fields such as pharmacy, medicine, industry, agriculture, consumer products, electronics, etc., are already present in our daily life, their potential risks and safety issues for human health are serious concerns for society [29,30,31]. Although 3D models are more interesting than 2D cell culture models for biomedical investigations [7,32], the application of spheroid models for nanotoxicity analysis is still in its infancy. The toxicity of nanomaterials, nanostructures, and nanotechnology-made products and also the nanoparticles penetrating cells is not only influenced by their physico-chemical properties such as size, type, shape, and surface charge [9,33], but also by the uptake and distribution in larger tissue structures. For instance, the surface charge and size of dendrimers exhibit as the major factors to maximize the therapeutic potential of loaded drugs by controlling their distributions to spheroid [34]. Consequently, optimization of cell migration, cell–matrix/cell interactions, NP-based cellular responses, reproducibility and scalability, compatibility with available analytical methods, dose-dependent cell viability, and interaction with nanomaterials should be addressed for 3D spheroid methods in nanotoxicology [7,16,24]. 

The current study sets out to provide a better understanding of how the uptake rate of AuNWs could be influenced by 3D multi-cellular spheroids. The use of nanomaterials as theranostic (therapy and diagnostic) tools promises advantages such as enhanced efficacy, better drug stability, biocompatibility or bioavailability, improved dosing, and reduced side effects [21,35,36]. Gold nanotoxicity has been studied by scientists due to its importance for biomedical applications, and it is well established and described for drug delivery platforms [37,38,39]. Based on our literature reviews, the toxicity of AuNWs had not been extensively evaluated by three-dimensional spheroids before and was not compared by 2D cell culture platforms. Therefore, in this study, the AuNWs’ toxicity was evaluated by three factors (metabolic activity, live/dead assay, and spheroid integrity) using two comparative 2D and 3D cell-based platforms. Inorganic nanowires including AuNWs have plenty of applications, such as: gene and biomolecular delivery; penetrated nanowires into cells for optical/electrical stimulation and recording; rare cancer cells capture, separation, and analysis; cell adhesion/spreading; and finally, mechanical tuning [40]. 

A comparative study of dose-time response relationships by 2D vs. 3D cell cultures platforms and using PC9 and A549 non-small lung cancer cells is conducted to address changes in cell viability, metabolic activity, and spheroid integrity. The aim of this work is to provide additional information on the impact of multi-cellular architectures on gold nanowire-induced toxicity by investigating three factors including the disrupting spheroids’ shape integrity, metabolic activities, and cell apoptosis (live/dead assay).

## 2. Materials and Methods

### 2.1. Gold Nanowire Synthesis and Characterization

The AuNWs were synthesized by the anisotropic elongation method as reported in our previous work [1,2]. Briefly, first, we had prepared two seed and growth solutions separately. For seed solution preparation, the chloroauric acid (HAuCl_4_ 0.25 mM) and sodium citrate (0.25 mM) (all from Sigma Aldrich, Merck KGaA, Darmstadt, Germany) were mixed in 20 mL double-distilled water followed by addition of 600 µL NaBH_4_ (0.1 M, Sigma Aldrich) in vigorous stirring. At this stage, the color of the mixture turned into a deep red. A spherical gold nanoparticle, 4 nm in size, was synthesized and it was kept at 4 °C (for a few months). The solution kept several hours at room temperature (RT) for evaporation of NaBH_4_ then stored in 4 °C. In the next step, the growth solution was prepared by adding 0.1 M of cetyltrimethylammonium-bromide (CTAB, Sigma Aldrich) to 200 mL deuterium-depleted water (DDW) and heated, with vigorous stirring, followed by adding HAuCl4 (0.25 mM) and 0.5 mM of ascorbic acid (Sigma Aldrich) to the CTAB solution. The color of the solution turned from deep yellow to pale yellow due to the Au (III) to Au (I) reduction. Finally, we added 70 mM of nitric acid (Sigma Aldrich) to the solution. For synthesis of the high aspect ratio of AuNWs, the solutions were aliquoted into three glass vials as follows: glass vials number one and two (25 mL separately), and glass vial number 3 (250 mL). The final growth solution was aliquoted between the mentioned glass vials as follows: 9 mL to number 1; 18 mL to glass number 2; and 173 mL into glass vial number 3. Under vigorous stirring, the growth of the gold nanowires started by adding 1 mL prepared seed solution to glass vial 1 and kept for 15–30 s. Then, 1 mL of the solution was transferred from glass vial one to glass vial two, then stirred, and kept for 30 s under vigorous stirring. At the final step, 5 mL of the solution from glass vial 2 was transferred to glass vial 3 under vigorous stirring and kept for 2 h at 37 °C. The 50 mL centrifuge tube was used to collect and save the solution for 1 week at 37 °C without stirring. A brown pellet at the bottom of the 50 mL centrifuge tube revealed well-synthesized AuNWs, and it contained 90% usable AuNWs. The supernatant was discarded and the pellet was re-suspended in DDW before use. The characterization methods such as field-emission scanning electron microscopy (FESEM, SEM-Model tech, KYKY Technology Development Ltd., Beijing, China), and ultraviolet–visible spectroscopy (UV-Vis, UV-Vis EU2200, OnLab, Shanghai, China) were applied to validate the formation of AuNWs.

### 2.2. Cell Culture and Spheroid Generation

Two different lung cancer cells, PC9 and A549, were used to evaluate nanotoxicity and uptake rate of AuNWs by two different cell systems including 3D multi-cellular spheroids and 2D monolayer cell culture platforms. The cell lines were grown in RPMI 1640 (Sigma-Aldrich, Vienna, Austria) supplemented with 10% fetal calf serum (Gibco, Thermo Fisher Scientific, Waltham, MA, USA), and 1% antibiotic/antimycotic solution (Sigma-Aldrich, Austria). The cells were cultivated in 25 and 75 cm^2^ cell culture flasks and kept in the incubator condition (37°C, 5% CO_2_, and 95% RH) till appropriate cell confluency. A Corning^®^ spheroid microplate’s 96-well plate were used for spheroid generation. To this end, as well as for 2D cell culture systems, after 80% cell confluency, it was trypsinized and the pellets were collected (1250 rpm for 5 min), before they were seeded at an initial cell concentration of 1.5 × 10^4^. For 2D cell culture systems, the cell concentrations were 1.5 × 10^4^, 10 × 10^3^, and 7 × 10^3^ cells per well for 24, 48, 72 h. After approximately 5–6 h, a spheroid was generated and cells were cultivated in a 2D monolayer (96-well plate). Both 3D and 2D systems were subjected to different concentrations of AuNWs (50, and 500 µg/mL and 2.5 mg/mL, respectively). 

### 2.3. Quantitative Live/Dead Assay

Based on Table 1, for the determination of metabolic activity and cell proliferation, a fluorescence PrestoBlue assay was used according to manufacture protocol (10:1 diluted with RPMI in the dark place). The cells media of each well-plate was discarded smoothly (for spheroids systems) and the media supplemented with PrestoBlue reagent was added to each well-plate, kept in a dark incubator for 2 h and the absorbance was recorded by using the plate reader (Ex: 570 and Em: 600). To quantify the metabolic activity (cell viability and cytotoxicity), the following formula was used:

(OD_test_  −  OD_blank_)/(OD_control_ − OD_blank_) × 100%

where OD_test_ is related to the absorbance of nanomaterial-treated samples, OD_blank_ is related to the absorbance of well-plate without cells, and OD_control_ is related to absorbance of nanomaterial-free samples.

Fluorescence LIVE/DEAD^®^ Viability/Cytotoxicity assay (Calcein-AM and EthD-1) was used for staining the live/dead cells in both 3D and 2D cell culture systems (Table 1). According to manufacturer protocols, the Calcein-AM and EthD-1 were added to the media, the cell images were taken by using an inverted fluorescence optical microscope (TE2000, Nikon, Yokohama, Japan) equipped with a digital camera (DS-Qi1MC, Nikon, Yokohama, Japan).

### 2.4. Metabolic Activity Measurement

The metabolic activity using PrestoBlue™ cell viability reagent was measured by the following manufacturer’s protocol. Prestoblue is an element that is rapidly reduced by the metabolic activity of active (live) cells. According to the manufacturer’s protocol, a 10:1 ratio solution was diluted with the medium in the dark place. Then, the previous medium was slowly discarded and 100–150 µL medium containing Prosto Blue was added to each well. The plate was kept in an incubator without light) for 2 h and then the absorbance was measured (Ex: 570 nm and Em: 600 nm) by the plate reader. Finally, according to the following formula, metabolic activity was calculated and cell growth and cell toxicity were estimated [3,17].

(OD_test_ − OD_blank_)/(OD_control_ − OD_blank_) × 100%

where: OD_test_, is the absorbance of NPs treated samples, OD_blank_ is the absorbance of free-cell samples, OD_control_ is the absorbance of non-NPs samples.

Finally, the data obtained were subjected to evaluation of the metabolic activity. One-way ANOVA and Tukey’s mean comparison test were used to compare the mean data at the level of 0.01% and 0.05%. 

### 2.5. Measurement of the Spheroids Surface Area 

For calculation of the spheroids’ sizes (surface area) and maintenance of the integrity of the spheroids’ architectures (morphology of spheroids) during NPs treatment, surface area index of the spheroids was used. Micrographs were taken by using an inverted fluorescence optical microscope (TE2000, Nikon, Japan) equipped with a digital camera (DS-Qi1MC, Nikon, Japan) on different days. The spheroid size was analyzed by ImageJ (Madison, WI., USA) software. To this end, the spheroid surface area was measured by using the BioVoxxel Image Processing and Analysis Toolbox for ImageJ (BioVoxxel, Ludwigshafen, Germany) [3]. It should be noted that this parameter was not used for the 2D monolayer. 

### 2.6. Statistical Analysis 

For live/dead assay and calculation of the spheroids’ surface areas, the fluorescence images of the spheroids and the monolayer cells data were collected from the microscopy technique and were utilized for the analysis of variance (ANOVA). In addition, the comparison of the mean using Duncan’s range test at *p* = 0.05 was analyzed. The means ± SE were used to compare the data. Mean fluorescence intensity of the spheroids and monolayer cells were analyzed pixel-by-pixel using Fiji (version 1.52i, Fiji, Madison, WI, USA). Furthermore, the GraphPad Prism software (version 8.0.2, Prism Corporation Pvt Ltd., San Diego, CA, USA) was used to design graphs and statistical analysis. 

## 3. Results and Discussion

The results of the gold nanowire characterization including field-emission scanning electron microscopy (FESEM), and ultraviolet–visible spectroscopy (UV-Vis) are reported in our previous works [1,2]. 

**Figure 1:**
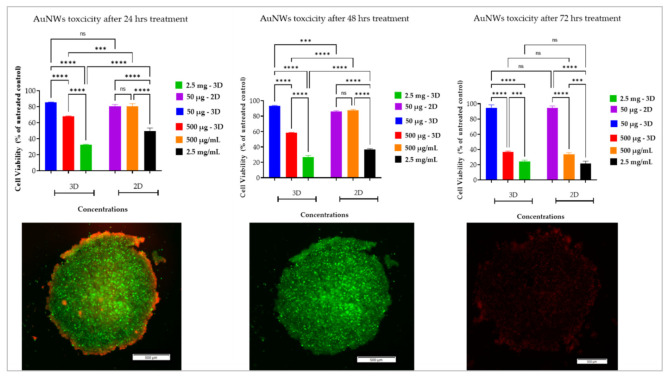
Live/dead assay of 3D multicellular spheroids and a 2D monolayer of A549 and PC9 lung cancer cells under treatments of different concentrations of AuNWs NPs evaluated by Calcein AM reagent: All AuNWs doses are per mL^−1^; Error bars represent ± SD (*n* = 3); ns is non-significance; *** *p* < 0.001; **** *p* < 0.0001 (highly significant); scale bar is 500 μm. The fluorescence images of A549 spheroids presented from right to left included dead cells stained with EthD-1, live cells stained with Calcein-AM, and merged.

To estimate the differences between 2D and 3D in vitro cell-based nanotoxicity assays, two different lung cancer cell lines A549 and PC9 are cultured either as a 2D monolayer or 3D multicellular spheroid and exposed with gold nanowire suspensions over 24, 48, and 72 h. The result of the live/dead assay accomplished by Calcein-AM and Ethidium Homodimer-1 (EthD-1) is shown in Figure 1. The result of cell viability revealed higher biological activities in 2D cell cultures platforms than 3D spheroids, pointing at increased proliferation rates or recovery of cell proliferation in 2D over 3D spheroid systems. 

The data of cell viability showed that, for example, the value of viability on the 2D cells’ platforms was 80%, while this value for the spheroids was approximately 60% (500 µg/mL at 24 h treatment), resulting in the spheroids being more affected by AuNWs or having a higher cell proliferation recovery. The incubation time (tolerant time of AuNWs by cells) and onset of internalization as well as concentration, seem be the most important factors in this case [41]. The cells, both in the 3D spheroids and 2D monolayer cell culture platforms, can tolerate a low dose of AuNWs at initial exposure time, but after a longer time (after 24 h) the cells have shown their cytotoxicity (at a low concentration). 

The calculated IC50 for the 2D cell cultures platform was 1937 µg/mL (at 24 h), 1604 µg/mL (at 48 h), and 218 µg/mL (at 72 h), while for 3D multicellular spheroids, it was 1326 µg/mL (at 24 h), 1176 µg/mL (at 24 h), and 411 µg/mL (at 24 h). These results were confirmed by another report [41], and they showed the onset of internalization in a similar manner. In addition, they showed that the molecular mechanisms of cellular uptake of nickel nanowires take place through the phagocytosis pathway. The results of fluorescence intensity revealed that the spheroids are more sensitive than cells that are grown in 2D monolayer platforms, but this sensitivity depends on the time-dose manner. In contrast, 2D cell monolayers showed a tendency to recover their proliferation from 500 µg/mL dosages. In addition, a significant toxicity difference is found between 2D monolayer and 3D spheroids in terms of concentration (dose-manner) and exposure time. Ni et al. [42] have developed different nano-formulations of polymeric nanodots (≈10 nm) and nanoparticles (≈70 nm), for penetrating efficiency study in cells. Their result showed that the size of nanocarriers has influenced the tumor cell uptake rate, resulting in the nanodot’s uptakes being higher than the nanoparticles containing an anticancer drug (paclitaxel). In contrast, they functionalized the nanocarriers (nanodots and nanoparticles) by iRGD peptides, and their results revealed that nanocarriers with different sizes but similar surface functionalization showed no significant difference in 2D cellular uptake but the uptake in multicellular tumors was size-dependent. The cell densities in the spheroids and tumors as natural diffusion barriers and other biological limitations such as increasing acidic microenvironment and hypoxia that occurred in the spheroid core zone makes the spheroid more resistant to the drug and biological molecules penetration [3]. The phenomenon in which the drug or nanoparticles are inhibited and their transport into deeper regions of the spheroid is restricted through physical limitations and also intercellular interactions are not available for 2D monolayer cell culture platforms [3,25,43]. 

Furthermore, a single dose of AuNWs has shown an ability to inhibit cell proliferation in 3D spheroids and effectively disrupt cell-to-cell interactions. Our results also revealed that the cellular responses to AuNWs stimuli were affected by cell-line type where the A549 cell line was more sensitive than PC9 in terms of cell toxicity caused by the gold nanowires. Understanding how different types of cells respond to nanoparticles, and finding information through what mechanisms the cells are affected by NPs is of paramount importance to the design and development of nanomaterials with high safety and efficacy. In a study, the cell types have shown different responses to NPs toxicity based on their physiological function and location [44]. In the case of live/dead assay, the results showed that the live cells (green fluorescence intensity) consistently show different responses in 2D and 3D spheroids. In turn, at higher AuNWs concentrations (e.g., 500 μg/mL and 2.5 mg/mL at 72 h), cell cultures showed lower/higher viability which suggests a higher potential to uptake the nanowires. It is important to note that fluorescence intensity corresponding to the live cells in the outer side of the spheroids decreased, while the deeper regions seemed unaffected, thus indicating AuNWs uptake by the surface of the spheroids and its limited transport inside the spheroid (Figure 1, fluorescence images of spheroids). The phenomenon of dose-time toxicity of 2D and 3D methodologies can be explained by several reasons, including: (a) drug/nanoparticle diffusivity into the spheroids is reduced; (b) the quiescence cells presence, as well as the occurrence of hypoxia in the core zone of the spheroids; (c) gene expression level alteration; (d) enhancement of cell–cell interactions; and (e) the presence of the extracellular matrix [3,45].

The Table 2 and Table 3 list corresponding metabolic activity data featuring the mean comparison in 2D monolayer and 3D multicellular spheroids in the presence of AuNWs. The metabolic activity fingerprint of the cells that have grown in the different growth conditions can adapt quickly to changes in nanotoxicity, chemical agents, and nutrient availability [5]. These results further indicate that the metabolism of the cells (metabolic activity) adapts to changes in the different concentrations of AuNWs and at different time exposure (time-dose manner). In turn, cells growing in 2D monolayers feature an exponential proliferation rate or recover their proliferation, resulting in increased metabolic activity under AuNWs treatment. However, the rate of cell proliferation in 3D spheroids does not follow an exponential growth rate due to geometric restrictions of the 3D environment. The proliferation of cells located in the core zone of the spheroids (depending on spheroid size) can be inhibited due to a lack of nutrients, O_2_, and higher concentrations of CO_2_ [4,17,46]. The metabolic activity of 3D platforms of the grown cells are comparable, in many cases, and mimics physiological microenvironments of tissue, as well as to cells directly isolated from in situ tissue [5]. More viable cells correspond to more metabolic activity; therefore, the value of the metabolic products at 24 h (not affected by AuNWs) is higher. At 48 and 72 h by onset of internalization due to the higher concentration, we witnessed more dead cells than live cells and, therefore, a decrease in the metabolic activity. For more incubation time and higher doses of AuNWs, it has been reported that the cells shift to the late necrotic/apoptotic [41]. A detailed description of the metabolic activity under different AuNWs concentrations and different times can be found in Table 2 and Table 3.

**Figure 2:**
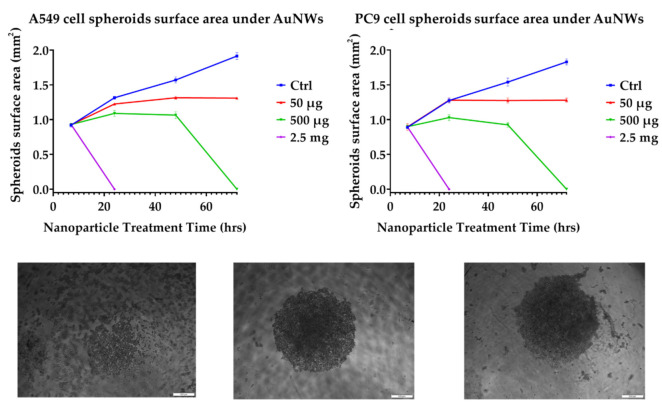
The surface area of 3D multicellular spheroids of A549 and PC9 lung cancer cells under treatment of different concentrations of AuNWs NPs: error bars represent ± SD (*n* = 3). The bright-field images of A549 spheroids from right to left include low integrity (cell–cell contacts) of spheroids treated to AuNWs, strong integrity (cell–cell contacts) of spheroids, and disrupted spheroids integrity (cell–cell contacts) of spheroids treated to AuNWs. The scale-bar is 500 μm.

Figure 2 shows the data of surface area changes of 3D multicellular spheroids under AuNWs treatment at different times. At high concentrations of AuNWs, AuNWs disrupt the spheroid’s integrity as they disconnect the cell -cell interaction. The same result was reported by another study, where they used Trans-Epithelial Electrical Resistance (TEER) assay to study the cell integrity under nanowire toxicity [41]. Although, the 3D spheroids integrity disrupting was affected by the AuNWs time-dose manner, but the data showed that it was not for all concentrations in the 3D spheroids platforms, indicating the fact that the spheroids can tolerate a lower dose of AuNWs. In fact, at low concentrations (50 μg/mL) the spheroid’s integrity was not affected by the AuNWs exposure time and concentration, but it was affected at high AuNWs concentrations (500 μg/mL and 2.5 mg/mL). The spheroid’s surface area increase, which is related to the cell proliferation, has a sigmoidal curve. In comparison to the control (without AuNWs), the samples treated with 50 μg/mL AuNWs showed a size increase of the spheroids until 24 h, and after that the size increase ceased (48 h). In this case, the spheroids did not lose their spherical shape even after 72 h exposure time, but the spheroid’s surface area increase came to an end. At higher concentrations, i.e., 500 μg/mL, the spheroid’s size increased at 24 h and then the integrity of spheroids was disrupted at 72 h. For both cell lines, the cell–cell interaction was disconnected and the spheroids lost their spherical shape during the first instance (24 h or before) of exposure to AuNWs. In the case of spheroid integrity and cell spheroid growth, especially in this study, we can hypothesize three effective manners under treatment of AuNWs. The first is growth and sigmoidal increasing of the spheroids’ surface area (no toxic effect in the control samples); the second manner is the termination of the spheroids’ growth but not the spheroids’ integrity loss (50 μg/mL); and finally disrupting the spheroids’ integrity and losing cell–cell interactions (at 72 h of 500 μg/mL and 2.5 mg/mL). 

Previously, 2D cell-based assays have been used as traditional systems to evaluate drug screening/discovery, nanotoxicity, etc. These platforms have numerous limitations and scientists have been looking for alternative platforms to mimic the real physiological properties of tissue and organs. Three-dimensional platforms, such as the spheroid system, can translate physiological functions of the tissue and organs of our body and the data acquired by 3D spheroids’ cell-based platforms, and are more realistic than 2D cell culture systems [47,48,49]. In case of lung cancer, targeted therapy of lung cancer is highly dependent on the identification of the molecular mechanisms of progression and development, as well as prognosis, to open new avenues in this case [50]. In this study, we have found that the results of different cell lines have significant differences in the case of nanotoxicity. The molecular subtypes of lung cancer are based on different genetic and cell-surface markers. Treatment (here, toxicity by AuNWs) of each subtype corresponds to molecular tests to identify the subtype and to suggest potential drugs to treat it [50]. Hence, different subtypes have different cell surface markers, thereby more or less drugs or nanoparticles (with different size, shape, type, etc.) can be taken up and finally influence the cytotoxicity. The difference in the live/dead rate of cells corresponds to different morphology, cell membrane, and cell cavity size [51]. The same results were reported by researchers in [44], where their results revealed that the nanomaterial’s size, composition, and cell type are critical factors of intracellular responses to nanomaterials, cytotoxicity rate, as well as potential mechanisms of toxicity. They claimed that variability of nanotoxicity between cell types contributes to the physiological function, which is different and related to cell types. The difference in the live/dead rate of the cells corresponds to the different morphology, cell membrane, and cell cavity size [51]. The same results were reported by others too [44], where their results revealed that the nanomaterial’s size, composition, and cell type are critical factors of intracellular responses to nanomaterials, cytotoxicity rate, as well as potential mechanisms of toxicity. They claimed that variability of nanotoxicity between cell types contributes to their physiological function, which is different and related to the cell types. In addition, the nanomaterials can be led to different intracellular responses due to the potential mechanism of toxicity. 

The results of another study suggested that the proliferation and mitosis of tumor cells treated with nanoparticles can be inhibited [52]. They claimed that the nanoparticles inhibited chromosome segregation but not chromosome replication, resulting in undivided giant nuclei. In the case of the metabolic fingerprint of cells grown under AuNWs treatment, the same results were reported by researchers where the metabolic activity of 3D cell culture platforms was less than 2D monolayers platforms [43]. Another study also revealed that the reactive oxygen species (ROS) of cells grown on 2D and 3D platforms have a significant difference, where 2D ROS was higher than 3D platforms [53]. The results of the spheroid surface area increase are not only dose-time dependent, but are also affected by the cell line; the A549 cell line is more sensitive in this case.

Cell-based assays, including those able to mimic physiological phenomena, are important because, for instance, stem cell therapies showed promise for treating various injuries and diseases [24]. The strategies that can improve the effectiveness of drugs and nanomaterials and decrease the observed therapeutic variability are necessary for biomedical applications. Recently, scientists generated differently sized stem cell spheroids by controlling the cell number and the cultivation parameters to the estimation of matrix components value, including fibronectin, laminin, and collagen [24]. Their results revealed that expression of the mentioned molecules and immunomodulatory factors significantly altered between the different sizes of the spheroids, corresponding to the metabolic activity of the spheroids. While the current research gives us good insight into the relative performance of AuNWs, this is still a primary step toward an understanding of how to fabricate and design more appropriate delivery systems for the tumors and also for biomedical applications. The results of a study revealed that penetration of nanowires into the cells is through the phagocytosis pathway, and cytotoxicity was increased after 24 h incubation time as well as by the addition of a dose of nanowires [41]. They used a lactate dehydrogenase (LDH) assay to measure the released LDH enzyme from cell cytoplasm due to losing the membrane integrity affected by the possible scenario that the nanowires are able to disrupt the cell membrane. Interestingly, the LDH level at 24 h for both treated and non-treated cells by nanowire was insignificant and therefore resulted in no cell membrane rupture, whereas in comparison to the cells not treated by the nanowires, LDH leakage was significantly increased at 48 and 72 h of nanowire treatment. In addition, to evaluate the cell integrity, they used TEER assay, and these results confirmed the LDH assay. In our study, it was revealed that the different spheroids generated by the different cell types are affected by the AuNWs in different manners. Different types of tumors will have different combinations of the matrix, and in addition, different host cells, with different characteristics that could influence their ECM and endocytosis rates [54]. Therefore, in future study, scientists’ emphasis may have to aim to develop different spheroid models comprising different tumor cell types such as, e.g., immune cells, fibroblasts cells, and cancer cells. This could help to improve our insights into how drugs, nano-encapsulated therapeutic agents, and nanoparticles penetrate the tumors. It should be noted also that the cell cultures (i.e., spheroids in our study) are in a homogeneous environment, whereas the tumor cells have a relatively heterogeneous one comprised of different types of cells and matrices.

In the case of nanoparticle penetration inside of the spheroids and tissue, researchers have claimed that this process can occur by different mechanism types such as convective or passive (diffusional), in addition to active cell mechanisms. Shape, size, surface charge, deformability, and other chemical/physical properties can influence these mechanisms. For example, polymeric particles can bend and are capable of deformations, while softer nanoparticles are able to circulate in the body for longer times in comparison to rigid nanoparticles (i.e., metal nanoparticles). In fact, particle deformability can be contributed to this phenomenon, which allows soft nanoparticles to ignore physiological barriers. This in turn led to the voyage of nanoparticles through blood vessels in the long term. The shape of nanoparticles can be attributed to their deformability potential. High aspect ratio nanoparticles have more deformability in comparison to low aspect ratio nanoparticles (same shape) [55], which is probably the most important reason for the penetration of AuNWs in the deep zone of the spheroids. Following treatment of nanoparticles into the cells, the intracellular generation of reactive oxygen species (ROS) overproduction by inducing ROS bursts [56,57]. This in turns lead to release of metal ions by nanoparticles which induces the ROS increase by mitochondrial respiration [58,59]. In the case of gold nanoparticles, an ROS-independent mechanism has been proposed by others for bacterial toxicity. They proposed molecular mechanisms underlying the toxicity and changes in some cell function that may occur includes chemotaxis enhancement, the decrease of F-type ATP synthase activities (decrease ATP level), a change (collapse) in membrane potential, and the inhibition of the subunit of ribosomes (decrease the tRNA binding) [57,60]. Overproduction of ROS in cells caused by metal nanoparticles can also induce oxidative stress. This could result in modification of proteins, gene expression modulation, lipid peroxidation, inflammatory responses, and redox activation of transcription factors, and in conclusion, these dysfunctions can lead to cell death [61]. 

## 4. Conclusions

An obvious need for a more reliable and comparable model to physiological phenomena similar to our 3D tissue and organs guided scientists to develop 3D cell-based assays. The 3D multicellular spheroid is an interesting cell-based assay for drug screening/testing and nanotoxicity evaluation, in addition to other cell-based assays. The biggest advantage of the 3D multicellular spheroids platforms in comparison to 2D cell culture methodologies is that they are able to mimic our body’s physiological conditions. Additionally, the spheroid integrity, metabolic fingerprint, and live/dead quantitative assay enabled us to learn how 3D and 2D are more affected by Au nanostructures’ toxicity. The investigation was carried out at different time-dose manners. It was noticed that the 3D spheroid integrity of lung cancer cells were more sensitive to the AuNWs toxicity than 2D cell culture treatments. One of the most important factors influencing the results of 2D and 3D spheroid changes is known as the ECM of solid tumors. The ECM is able to act as a transport barrier that moderates NPs penetration into the spheroids. In another work, the researchers had evaluated the effects of NPs size and collagenase enzyme treatment of the spheroids on the penetration of fabricated NPs in a 3D multicellular spheroid model [62]. NP penetration was not only impacted by their size into the spheroid, but it also was influenced by the treatment of the spheroids by collagenase enzyme. In fact, the collagenase can degrade collagen as a supporter of the spheroids and barriers of NPs penetration. In our study, it was found that the results of the live/dead assay and metabolic activities of 3D and 2D are significantly different after AuNWs treatment and are influenced by dose concentrations and exposure time. Our results confirmed that 3D multicellular spheroids could be a good model/tool for optimizing and evaluating nanomaterial toxicity and anticancer therapy parameters. These findings also demonstrate that degradation of the ECM by AuNWs and other agents can significantly enhance the efficacy of numerous therapeutic systems, such as drug-loaded nanocarriers in solid tumors. In addition, the results of this study can help us to develop more reliable cell-based assays similar to the spheroids as an alternative to animal testing. One of the most probable mechanisms of cytotoxicity caused by nanoparticles is reactive oxygen species (ROS) generation [61]. Accelerating the use of nanotechnology-made products in the biomedical field is heavily dependent to their biosafety improvement, therefore some challenges need to be overcome. First, the need for high-throughput evaluating techniques should be developed to examine the nanotoxicity of nanomaterials in in vitro and in vivo like methods similar to our study. These types of techniques are able to save time and resources, as well as translate all-in-one systems in which multiple assays are handled in a single system, and decrease methodological and systematic errors [63]. Second, the molecular/cellular mechanisms of ROS generated by nanoparticles must be clear to help us to providing information and a deep understanding of how to modify the physico-chemical properties of nanoparticles to control ROS generation. In short, these will provide new insights for scientists to create novel strategies to increase the safety of engineered nanomaterials to be used in different applications, including the clinical, commercial, and biomedical fields. 

## Figures and Tables

**Table 1:** The chemical reagent and their details were used in this study.

Activity	Chemical Reagent	Company
Spheroid generation microplate	Unknown	Corning^®^ spheroid microplates 96 well
Live cells staining	Calcein-AM	Calbiochem (Merck Biosciences)
Dead cells staining	Ethidium Homodimer-1 (EthD-1)	Invitrogen, Thermo Scientific
Metabolic activity and cell proliferation	PrestoBlue Cell Viability Reagent	Invitrogen, Thermo Scientific

**Table 2:** Comparison of metabolic activity of 3D and 2D systems of A549 cancer cells treated with different concentrations of AuNWs at different times.

	2D Monolayer		
Conc. and Time	2.5 mg/mL	500 μg/mL	50 μg/mL
24 h	** 58.89 ± 1.03	** 70.4 ± 3.33	^ns^ 98.3 ± 2.1
48 h	** 11.5 ± 4.22	* 74.3 ± 0.19	^ns^ 96.4 ± 1.09
72 h	** 7.5 ± 2.65	** 27.5 ± 2.34	** 84.23 ± 3.09
	**3D Multicellular Spheroid**		
24 h	** 45.52 ± 3.76	** 88.74 ± 2.44	^ns^ 98.42 ± 2.87
48 h	** 18 ± 2.5	** 68.98 ± 3.87	^ns^ 97.7 ± 2.47
72 h	** 0 ± 0	** 24.4 ± 0.89	** 76.01 ± 2.49

Data are shown based on RFU: relative fluorescence intensity. Cell viability is directly related to metabolic activity and ultimately RFU. The quantitative results are the mean reduction of Resazurin reagent and the error bars represent ± SD (*n* = 3), ns is non-significance, * *p* < 0.05, ** *p* < 0.01.

**Table 3:** Comparison of metabolic activity of 3D and 2D systems of PC9 cancer cells treated with different concentrations of AuNWs at different times.

	2D Monolayer		
Conc. and Time	2.5 mg/mL	500 μg/mL	50 μg/mL
24 h	** 57.1 ± 1	** 82.1 ± 1.34	^ns^ 96.6 ± 1.7
48 h	** 14.2 ± 2.09	* 83.21 ±1.44	^ns^ 99.55 ± 2.4
72 h	** 6.02 ± 1.14	** 22 ± 2.4	** 77.589± 1.9
	**3D Multicellular Spheroid**		
24 h	** 40.46 ± 2.93	** 81.3 ± 1.26	^ns^ 93.2 ± 1.14
48 h	** 17.6 ± 4.09	** 66.6 ± 1.51	^ns^ 98.43 ± 2.07
72 h	** 0 ± 0	** 20.22 ± 2.35	** 71.31 ± 1.3

Data are shown based on RFU: relative fluorescence intensity. Cell viability is directly related to metabolic activity and ultimately RFU. The quantitative results are the mean reduction of Resazurin reagent and the error bars represent ± SD (*n* = 3), ns is non-significance, * *p* < 0.05, ** *p* < 0.01.

## Data Availability

Data are available upon request.
